# Onset of the Thermic Effect of Feeding (TEF): a randomized cross-over trial

**DOI:** 10.1186/1550-2783-4-24

**Published:** 2007-12-05

**Authors:** Christopher B Scott, Jill Fernandes, Maya Lehman

**Affiliations:** 1 University of Southern Maine; Exercise, Health and Sports Sciences, Gorham, ME, USA

## Abstract

**Background:**

The purpose of this investigation was to identify the onset of the thermic effect of feeding (TEF) after ingestion of a high carbohydrate (CHO) and a high protein (PRO) 1255 kJ (300 kcal) drink.

**Methods:**

Resting metabolic rate (RMR) and TEF were measured over 30-minute periods via indirect calorimetry using a ventilated hood technique. Eighteen subjects (7 men and 11 women) completed two randomized, double-blind trials. Data were collected in 1-minute measurement intervals. RMR was subtracted from TEF and the time of onset was obtained when two consecutive data points exceeded 5% and 10% of resting metabolic rate.

**Results:**

At 5% above RMR the onset of TEF for CHO was 8.4 ± 6.2 minutes and was not different as compared to PRO, 8.6 ± 5.2 minutes (p = 0.77). Likewise, no differences were found with a 10% increase above RMR: CHO, 14.1 ± 7.5 min; PRO, 16.7 ± 6.7 min (p = 0.36). Several subjects did not show a 10% increase within 30-min.

**Conclusion:**

We conclude that the onset of TEF is variable among subjects but is initiated within about 5 to 20-min for most subjects after ingestion of a 1255 kJ liquid meal. No differences were found between CHO or PRO liquid meals.

## Background

Antoine Lavosier (1743–1794) may have been the first to directly connect digestion with an increased heat production and oxygen consumption [1]. Later in the 20^th ^century it was discovered that increases in energy expenditure after eating, called the thermic effect of feeding (TEF), varied depending on the composition of the meal [2]. Metabolic rate not only rises to a greater extent with an increased caloric intake [3,4] but there is a 6–8% increase in energy expenditure with carbohydrate meals, 3% increase with fat, and 25–40% with protein based meals [2]. Research tends to focus on the extent and timing of the peak TEF where measurements can go on for hours [5]. Measurement intervals too can be lengthy often occurring in 15 or 30-minute periods. Lengthy measurement intervals reduce measurement variability [6] but they also make it difficult to pinpoint precise time periods; for example, determining when TEF rises.

Sports nutritionists have brought timing issues to attention where it has been recommended that nutrient ingestion post-exercise (i.e., recovery) be completed sooner rather then later to optimize nutrient uptake by muscle [7]. Likewise, it is apparent that both colder drinks [8] and lower drink volumes (meal size) [9] tend to hasten the gastric emptying time of a liquid meal. Dependent on the meal consumed blood glucose levels often rise within 10 or 15-minutes but again, measurement periods are often conducted in 10 to 15-minute intervals [10,11]. In this regard it is not known if the rise in blood glucose above fasting levels is meaningful or not within the first measurement period.

In this investigation we asked the following 'timing' questions: 1) at a given 5% and 10% increase in RMR, when exactly does energy expenditure begin to rise after feeding and, 2) is the onset of TEF different between equi-caloric carbohydrate and protein liquid meals (1255 kJ; 300 kcal)? Comparisons between sexes also were made.

## Methods

### Subjects

Subjects consisted of eighteen healthy participants (males, n = 7; females, n = 11) (see Table 1): age, 25.9 ± 9.1 years; height, 169.9 ± 9.1 cm; weight, 67.2 ± 11.4 kg. Prior to testing subjects read and signed an informed consent. All procedures were approved by the Institutional Review Board (IRB) of the University of Southern Maine.

**Table 1:** Subject characteristics (mean ± SD)

	Men (N = 7)	Women (N = 11)	p	All Subjects (N = 18)
Age	24.9 ± 9.0	26.5 ± 9.6	0.65	25.9 ± 9.1
Height (cm)	178.4 ± 8.3	164.7 ± 5.1	0.009	169.9 ± 9.1
Weight (kg)	78.0 ± 9.4	60.3 ± 5.8	0.001	67.2 ± 11.4
RMR CHO (ml O_2 _min^-1^)	352.9 ± 50.9 a	291.8 ± 41.9 b	0.02	315.6 ± 53.7 c
RMR PRO (ml O_2 _min^-1^)	365.7 ± 42.8 a	274.5 ± 35.6 b	0.001	310.0 ± 59.0 c

RMR = resting metabolic rate; CHO = high carbohydrate; PRO = high protein.

p = t-test alpha level of between sexes comparisons

a, b, c represent t-test comparisons between CHO and PRO:

a, p = 0.62; b, p = 0.31; c, p = 0.77

### Procedures

Metabolic data were collected using a ventilated hood (Parvo Medics; Sandy UT). Previous investigations have shown the ventilated hood method to be more accurate and reliable when compared to the other techniques [12,13]. This method also has proved to be more comfortable in tests that last more than five to ten minutes [12].

Participation in this study entailed two separate visits to the Human Performance Lab at the University of Southern Maine (Gorham campus); a different drink was randomly provided at each visit. Subjects were encouraged to report to each visit at the same time of day to ensure consistency. Testing occurred between 8:00 AM to 12:00 PM. Each test required a three hour fast (including water consumption). Exercise was not allowed on the day prior to testing. On both testing days, subjects lay comfortably in a semi-recumbent position on a gurney for 60-minutes and were encouraged to position themselves in a way that would refrain from fidgeting. Resting gas exchange measurements were collected using a Parvo Medics metabolic cart (Sandy, UT); the cart was calibrated for gas analyses and volume at least twice prior to each test. Room air (25°C) was drawn through the hood at a rate of 40 L min^-1^. Data were recorded each minute.

Resting metabolic rates (RMR) were collected over the initial 30-minute reclining period with the last 10-minutes averaged into an estimate of RMR. At 30-minutes the subject was given a 12-ounce, 1255 kJ, vanilla flavored liquid meal in a double-blind and randomized fashion: high carbohydrate (CHO) GNC Pro-Performance, Weight Gainer 2200 Gold (88% CHO, 10.9% PRO, 1.2% FAT) and high protein (PRO) Instantized Natural Whey (100%) Gold Standard (15.4% CHO, 73.8% PRO, 10.4% FAT). Both powders were mixed via blender with 12 oz of tap water (23°C). A 2-minute time frame was provided to consume each drink, after which data collection continued for another 30 minutes to identify the onset of TEF. Subjects were not allowed to watch TV, talk, or fall asleep during the tests.

After ingestion subjects assumed a reclining position. The first 2-minutes of data collection post-ingestion were not considered to be consecutive measurements (but minutes 3 and 4 were).

### Statistical Analyses

The onset of TEF was identified by two consecutive 1-minute measurement intervals that exceeded 5% and 10% of each individual's RMR. Gender and liquid meal comparisons also were made. Standard t-tests were used to determine differences between groups. Statistical significance was set at p < 0.05.

## Results

All data are reported as mean ± standard deviation. Subject characteristics for RMR are reported in Table 1: Men had a higher RMR as compared to women for both trials (p < 0.02). No differences in RMR were found between trials within males (p = 0.64), females (p = 0.24) or all subjects (p = 0.77) (Table 1).

For all subjects there were no significant differences in the onset of TEF between the CHO and PRO liquid meals when examined as either a 5% or 10% increase above RMR: 5% CHO, 8.4 ± 6.2 min; 5% PRO, 8.6 ± 5.2 min (p = 0.77); 10% CHO, 14.1 ± 7.5 min; 10% PRO, 16.7 ± 6.7 min (p = 0.36) (Table 2). Several subjects did not show any increase in TEF over the 30-min measurement period: 2 men never had a 5% increase in TEF with PRO; 1 man, 4 women had no 10% increase in TEF with CHO; 3 men, 1 woman had no 10% increase in TEF with PRO (Table 2).

**Table 2:** Onset of the thermic effect of feeding (mean ± SD)

	Men	Women	p	Men & Women	No Increase
5% Time (min) CHO	4.7 ± 2.9 a	10.8 ± 6.6 b	0.04	8.4 ± 6.2 c	0
5% Time (min) PRO	8.0 ± 4.2 a	8.8 ± 2.9 b	0.78	8.6 ± 5.2 c	2 male
10% Time (min) CHO	12.0 ± 7.7 d	15.9 ± 7.5 e	0.38	14.1 ± 7.5 f	1 male, 4 female
10% Time (min) PRO	18.8 ± 6.9 d	16.3 ± 6.9 e	0.57	16.7 ± 6.7 f	3 male, 1 female

5% time = onset of TEF that exceeds 5% increase in RMR; 10% time = onset of TEF that exceeds 10% increase in RMR; p = between sexes t-test p value; No Increase = no increase in TEF within 30-min was seen that exceeded 5% or 10% of RMR.

CHO = high carbohydrate; PRO = high protein.

a, b, c, d, e, f represent t-test comparisons between CHO and PRO:

a, p = 0.14; b, p = 0.46; c, p = 0.77; d, p = 0.20; e, p = 0.90; f, p = 0.36.

Between sex differences were evident for the initial 5% increase after CHO consumption in men (4.7 ± 2.9 min) as compared to women (10.8 ± 6.6 min) (p = 0.04). No other comparisons revealed significance between the sexes (Table 2).

## Discussion

**Figure 1:**
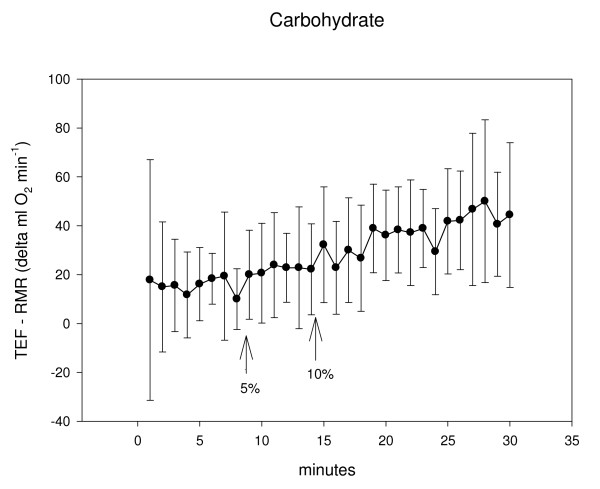
Metabolic rate is shown as RMR subtracted from TEF (Δml O_2 _min^-1^) in 1-minute intervals after ingestion of a 1255 kJ, high CHO liquid meal. The onset of TEF occurred at 8.4 ± 6.2 minutes for the 5% increase and 14.1 ± 7.5 minutes for the 10% increase. The large standard deviation for the initial data point is likely the result of the 2-minute consumption period where the ventilated hood was removed, subjects sat upright and the drink was ingested.

**Figure 2:**
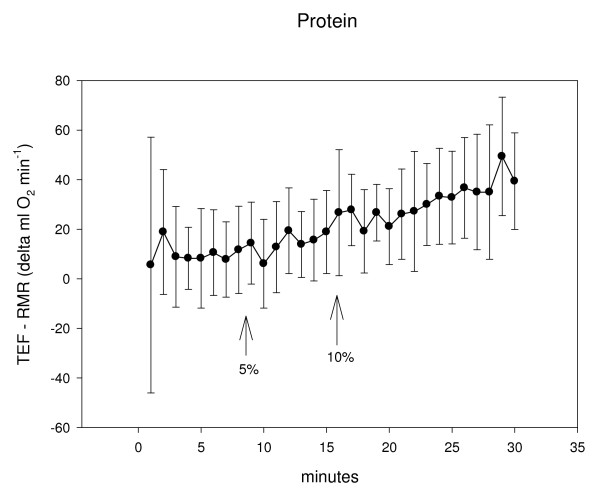
Metabolic rate is shown as RMR subtracted from TEF (Δml O_2 _min^-1^) in 1-minute intervals for a 1255 kJ, high PRO liquid meal. The onset of TEF was found to occur at 8.6 ± 5.2 minutes for the 5% increase and 16.7 ± 6.7 minutes for the 10% increase. The large standard deviation for the initial data point is likely the result of the 2-minute consumption period where the ventilated hood was removed, subjects sat upright and the drink was ingested.

Previous research has demonstrated the timing of gastric emptying after ingestion of a liquid meal, variability is evident [8,9]. Our study was designed to determine the onset of the TEF. These results indicate that for a compilation of male and female subjects and regardless of whether the (room temperature, 1255 kJ) liquid meal is predominantly carbohydrate or protein, a 5% rise in energy expenditure after feeding occurs in about 8-minutes; a 10% increase in energy expenditure occurs in almost double that time at about 14–16 minutes. It is apparent that subject variability was evident, perhaps in accordance with the considerable between-subject variability that has been found with gastric emptying [8,9] (see standard deviations in Figures 1 and 2).

This investigation has limitations. Given between-subject variability, subjects could have been classified into specific groups for analyses, for example: comparing age, activity level, or body composition. A more direct investigation may better correlate gastric emptying with TEF. Our methodology also is subject to interpretation. For example, we considered the origin of the TEF as the first of 2 consecutive data points rising 5% and 10% above RMR. Perhaps 3 consecutive data points should have been chosen. Or perhaps the onset of TEF could better be depicted by a different statistical analysis (dependent on a measure of variance or being significantly different from a RMR baseline). We chose a 5% increase in energy expenditure based on the statistical alpha level where significance is found at p < 0.05. Some subjects never achieved 2 consecutive 1-minute rises in energy expenditure: 2 men for 5% increase with PRO, 3 men and 1 women for 10% increase with PRO, 1 man and 4 women for 10% increase with CHO (Table 2). Post-feeding measurements could have been extended to ensure a TEF onset with all participants (a pilot study of 3 subjects provided the rationale for our protocol revealing an observable increase in energy expenditure in less than 10-minutes). These data could also be affected by a limited 3-hour fast, the size (kcal and grams), temperature and type (solid versus liquid) of meal consumed and perhaps the amount of time spent consuming and the palatability of the meal [see [8,9,14]]. Female subjects in the current study were not tested according to their menstruation pattern. Research has shown that the phases of menses do [15] and do not [16] affect RMR and the TEF. In the carefully designed study of Tai et al. where TEF decreased during the luteal phase of menstruation, measurement times were conducted in 30-min intervals [15]; in our investigation onset time was recorded in 1-minute intervals throughout a 30-min period. Another factor that may have affected our results was the subject's anticipation and added movement after removing the hood for consumption of the drink. This resulted in considerable variation (standard deviation) for the first minute of TEF measurements (the first 2-min of TEF data were not considered as consecutive time points) (see Figures 1 and 2). Extended measurement times ensure less variability but our 1-minute collection periods were designed to help pinpoint the rise in the TEF.

Tai et al. postulated that glucose uptake was slowed during the luteal phase of menstruation suggesting the likelihood of a gender effect. Gender differences were only evident for the initial 5% increase in TEF with the CHO drink where women had a significantly lengthier onset of TEF as compared to men. A larger sample size will be needed to verify this. When using a 10% increase in TEF as a marker of significance, no gender or drink related (CHO vs PRO) differences were found.

Previous research has revealed a rather high degree of individual variability in gastric emptying [8,9] and the time to peak increase in TEF [5]. We conclude that the onset of TEF is likewise variable among subjects but is initiated within about 5 to 20-min for most subjects after ingestion of a 1255 kJ (300 kcal) liquid meal. The onset of TEF does not appear to differ between carbohydrate or protein liquid meals.

## Competing interests

The author(s) declare that they have no competing interests.

## Authors' contributions

C.B. Scott conceived and designed the study, performed statistical analysis and wrote the final manuscript. J. Fernandes and M. Lehman conceived and designed the study and collected all data. The authors have read and approve of this manuscript.
